# Efficacy of the quick sequential organ failure assessment for predicting clinical outcomes among community-acquired pneumonia patients presenting in the emergency department

**DOI:** 10.1186/s12879-020-05044-0

**Published:** 2020-04-29

**Authors:** Xiangqun Zhang, Bo Liu, Yugeng Liu, Lijuan Ma, Hong Zeng

**Affiliations:** Department of Emergency, Beijing Chao-Yang Hospital, Capital Medical University, No.5 Jingyuan Road, Shijingshan District, Beijing, 100048 P.R. China

**Keywords:** Quick sequential organ failure assessment, Community-acquired pneumonia, ICU-admission, Acute respiratory distress syndrome, 28-day mortality

## Abstract

**Background:**

The study aimed to investigate the predictive value of the quick sequential organ failure assessment (qSOFA) for clinical outcomes in emergency patients with community-acquired pneumonia (CAP).

**Methods:**

A total of 742 CAP cases from the emergency department (ED) were enrolled in this study. The scoring systems including the qSOFA, SOFA and CURB-65 (confusion, urea, respiratory rate, blood pressure and age) were used to predict the prognostic outcomes of CAP in ICU-admission, acute respiratory distress syndrome (ARDS) and 28-day mortality. According to the area under the curve (AUC) of the receiver operating characteristic (ROC) curves, the accuracies of prediction of the scoring systems were analyzed among CAP patients.

**Results:**

The AUC values of the qSOFA, SOFA and CURB-65 scores for ICU-admission among CAP patients were 0.712 (95%CI: 0.678–0.745, *P* < 0.001), 0.744 (95%CI: 0.711–0.775, *P* < 0.001) and 0.705 (95%CI: 0.671–0.738, *P* < 0.001), respectively. For ARDS, the AUC values of the qSOFA, SOFA and CURB-65 scores were 0.730 (95%CI: 0.697–0.762, *P* < 0.001), 0.724 (95%CI: 0.690–0.756, *P* < 0.001) and 0.749 (95%CI: 0.716–0.780, *P* < 0.001), respectively. After 28 days of follow-up, the AUC values of the qSOFA, SOFA and CURB-65 scores for 28-day mortality were 0.602 (95%CI: 0.566–0.638, *P* < 0.001), 0.587 (95%CI: 0.551–0.623, *P* < 0.001) and 0.614 (95%CI: 0.577–0.649, *P* < 0.001) in turn. There were no statistical differences between qSOFA and SOFA scores for predicting ICU-admission (Z = 1.482, *P* = 0.138), ARDS (Z = 0.321, *P* = 0.748) and 28-day mortality (Z = 0.573, *P* = 0.567). Moreover, we found no differences to predict the ICU-admission (Z = 0.370, *P* = 0.712), ARDS (Z = 0.900, *P* = 0.368) and 28-day mortality (Z = 0.768, *P* = 0.442) using qSOFA or CURB-65 scores.

**Conclusion:**

qSOFA was not inferior to SOFA or CURB-65 scores in predicting the ICU-admission, ARDS and 28-day mortality of patients presenting in the ED with CAP.

## Background

Community-acquired pneumonia (CAP) is a common infectious disease with high morbidity, mortality and medical costs [1], and is caused by various microorganisms such as bacteria, viruses, chlamydia and mycoplasma outside the hospital. Previous studies reported that approximately 20% of CAP adult patients needed to be hospitalized for treatment, and the mortality is as high as 30–50% [2]. The incidence of severe CAP needing admission to intensive care unit (ICU) is gradually rising due to septic shock and the requirement of invasive mechanical ventilation (IMV) [3]. The evidence has shown that severe CAP can induce the occurrence of acute respiratory distress syndrome (ARDS) which is a life-threatening disease with a present mortality of nearly 40% [4]. Therefore, it is significant for the clinicians to early predict the outcomes of CAP, accurately and objectively assess the severity of the disease to optimize therapeutic strategies.

To date, several assessment tools for CAP patients have been applied in the emergency department (ED), such as the quick sequential organ failure assessment (qSOFA) [5, 6], sequential organ failure assessment (SOFA) [7, 8], and confusion, urea, respiratory rate, blood pressure and age (CURB-65) scores [9–12]. Of these, the qSOFA score is recently developed to predict the death of infected patients which was proposed by the Sepsis-3 group in 2016 [13]. The advantage of the qSOFA score is on the basis of three clinical criteria (respiratory rate, mental status and blood pressure), without the requirement for laboratory tests. To the best of our knowledge, however, the predictive efficacy of these scoring systems on the pneumonia severity has rarely reported among patients with CAP.

Although some reports suggested that the qSOFA score is effective for predicting the mortality in CAP [14, 15], it is uncertain whether the qSOFA score can be used to evaluate other prognostic outcomes, such as ICU-admission and ARDS. Accordingly, we assessed the predictive value of qSOFA score for clinical outcomes in emergency patients with CAP, and the efficacy of the qSOFA score was explored in comparison with other pneumonia severity scoring systems (SOFA and CURB-65 scores) in ICU-admission, ARDS and 28-day mortality.

## Methods

### Patients

This investigation was conducted at Beijing Chao-Yang Hospital from Nov. 2011 to Sep. 2018. A total of 742 CAP cases were enrolled, containing 462 males and 280 females. The characteristics of patients were recorded including age, gender, past medical history and vital signs. The whole blood leukocyte counts, blood gas indexes, blood biochemistry and X-ray results were detected within 24 h. According to the findings of vital signs and laboratory examination, the qSOFA, SOFA and CURB-65 scores were calculated.

### Inclusion and exclusion criteria

Patients who met the following criteria were included: (1) age > 18 years; (2) corresponding to diagnosis criteria of CAP.

Exclusion criteria were: (1) advanced diseases including malignant tumors (advanced or metastasized tumors), end-stage liver or renal disease; (2) hospitalization within 14 days before symptom appearance; (3) cystic fibrosis, active pulmonary tuberculosis, severe immunosuppression, coagulopathy or systemic anticoagulant treatment; (4) pretreatment outside the hospital; and (5) the patients or relatives did not agree to participate in the study.

### Diagnosis criteria

Diagnosis criteria for patients with CAP conformed to new infiltration on the chest with at least one of the following symptoms: (1) cough; (2) sputum; (3) dyspnea; (4) core body temperature > 38.0 °C; (5) auscultation with abnormal breath sounds or rales [16].

Berlin diagnostic criteria for ARDS were utilized on the basis of oxygenation index as follows: (1) mild: 200 mmHg < PaO_2_/FIO_2_ ≤ 300 mmHg; (2) moderate: 100 mmHg < PaO_2_/FIO_2_ ≤ 200 mmHg; (3) severe: PaO_2_/FIO_2_ ≤ 100 mmHg. Four ancillary indicators for severe ARDS were considered including radiographic severity, respiratory system compliance (≤40 mL/cm H_2_O), positive end-expiratory pressure (PEEP, ≥10 cm H_2_O) and corrected exhaled minute volume (≥10 L/min) [17].

In the present study, we conducted a 28-day follow-up research to assess the outcomes. The occurrence of ARDS and 28-day mortality were served as the endpoint events, and ICU admission was also included as an outcome of patients within 72 h of ED visit. Patients who survived after the last follow-up were considered as survivors.

### Laboratory examination

Hospitalization within 12 h, 5–10 mL blood was collected in the tubes containing heparin or ethylenediaminetetraacetate (EDTA), then stored at − 80 °C. White blood cell (WBC) were measured via an automated hematology analyzer (Sysmex XS-500i, Sysmex Corporation Kobe, Japan). Plasma lactate (Lac) levels were detected by a blood gas analyzer (GEM Premier 3000, Instrumentation Laboratory, Lexington, MA, USA), and the normal range was 0.7–2.5 mmol/L. The concentrations of serum procalcitonin (PCT) were measured using a BioMerieux Mini VIDAS immunoassay analyzer (Block Scientific, Bohemia, NY, USA), and the limit of detection (LOD) was 0.05 ng/mL. Serum C-reactive protein (CRP) concentrations were analyzed utilizing turbidimetric immunoassay (BNII, Siemens Healthcare Diagnostic, Germany).

### Statistical analysis

Statistical analysis was performed using SPSS 16.0 (SPSS Inc., Chicago, IL, USA). Normally distributed data were expressed by mean ± standard deviation (SD), while non-normally distributed data were denoted via median (P_25_, P_75_). Mann-Whitney U test was used for the comparison between the two groups, and Kruskal-Wallis one-way analysis was utilized for the multi-group comparisons. Lac, PCT, WBC, CRP, qSOFA, SOFA and CURB-65 scores were analyzed in ICU-admission, ARDS and 28-day mortality, and we mentioned the receiver operating characteristic (ROC) curves and determined the area under curves (AUC). We also calculated the sensitivity, specificity, positive predictive value (PPV) and negative predictive value (NPV). Compared with AUCs, the formula of Z test is: $\mathrm{Z}=\left({A}_1-{A}_2\right)/{\left({SE}_1^2+{SE}_2^2-\mathrm{r}{SE}_1{SE}_2\right)}^{1/2}$ (Z_0.05_ = 1.96, Z_0.01_ = 2.58). The independent predictors of ICU-admission, ARDS and 28-day mortality were confirmed by Binary Logistic regression analysis. All statistical analyses were the two-tailed tests, and *P* < 0.05 was considered statistically significant.

## Results

### The baseline data of the emergency patients with CAP

A total of 1028 patients from ED were enrolled in this study. Among these subjects, 152 patients with incomplete clinical data had other diseases except CAP, 61 participants were lost to follow-up, and 73 patients or relatives did not agree to be enrolled. Finally, 742 patients completed 28 days of follow-up. The baseline data of the patients with CAP were shown in Table 1.

**Table 1:** The baseline data of the patients with CAP

Characteristics	ICU (*n* = 174)	Non-ICU (*n* = 568)	*P*	ARDS (*n* = 164)	Non-ARDS (*n* = 578)	*P*	Survivor (*n* = 443)	Non-Survivors (*n* = 299)	*P*
Age, years	70.40 ± 13.46	68.60 ± 13.74	0.128	70.26 ± 14.19	68.67 ± 13.53	0.189	68.23 ± 13.50	70.19 ± 13.90	0.055
Male, %	106 (60.92)	356 (62.68)	0.676	100 (60.98)	362 (62.63)	0.670	268 (60.50)	194 (64.88)	0.227
COPD, %	56 (32.18)	121 (21.30)	0.003	46 (28.05)	131 (22.66)	0.153	90 (20.32)	87 (29.10)	0.006
CVD, %	39 (22.41)	91 (16.02)	0.052	34 (20.73)	96 (16.61)	0.220	78 (17.61)	52 (17.39)	0.940
Hypertension, %	89 (51.15)	254 (44.72)	0.137	79 (48.17)	264 (45.67)	0.572	205 (46.28)	138 (46.15)	0.974
DM, %	68 (39.08)	190 (33.45)	0.173	65 (39.63)	193 (33.39)	0.138	136 (30.70)	122 (40.80)	0.005
CHF, %	86 (49.43)	236 (41.55)	0.067	72 (43.90)	250 (43.25)	0.882	180 (40.63)	142 (47.49)	0.064
CRD, %	27 (15.52)	57 (10.04)	0.046	20 (12.20)	64 (11.07)	0.689	46 (10.38)	38 (12.71)	0.327
Tumor, %	23 (13.22)	55 (9.68)	0.183	24 (14.63)	54 (9.34)	0.051	35 (7.90)	43 (14.38)	0.005
MBP, mmHg	81.91 ± 24.19	92.33 ± 18.88	< 0.001	82.34 ± 23.99	92.02 ± 19.16	< 0.001	92.15 ± 19.83	86.53 ± 21.54	< 0.001
Respiratory rate, beats/min	31.95 ± 7.33	29.72 ± 6.46	< 0.001	32.15 ± 8.30	29.70 ± 6.13	< 0.001	29.91 ± 6.27	30.74 ± 7.37	0.110
Temperature, °C	37.48 ± 1.30	37.41 ± 1.13	0.535	37.69 ± 1.36	37.35 ± 1.10	0.004	37.46 ± 1.14	37.37 ± 1.22	0.305
Heart rate, beats/min	78.11 ± 21.31	107.20 ± 31.20	< 0.001	116.60 ± 21.69	111.70 ± 29.41	0.079	109.30 ± 21.25	115.00 ± 29.64	0.002
PaO_2_, mmHg	73.54 ± 34.78	84.15 ± 31.45	< 0.001	66.49 ± 29.17	85.97 ± 32.19	< 0.001	81.92 ± 28.89	81.29 ± 37.37	0.804
Lac, mmol/L	4.47 ± 3.69	2.07 ± 2.05	< 0.001	2.60 (1.50,5.05)	1.50 (0.90,2.50)	< 0.001	1.50 (1.00,2.40)	2.00 (1.10,4.20)	< 0.001
PCT, ng/mL	1.50 (0.28,8.50)	0.28 (0.06,1.75)	< 0.001	1.62 (0.35,9.53)	0.27 (0.06,1.68)	< 0.001	0.24 (0.06,1.44)	1.06 (0.16,6.16)	< 0.001
WBC, ×10^9^/L	12.77 (8.23,18.97)	11.22 (7.99,15.66)	< 0.001	12.70 (7.73,17.67)	11.34 (8.11,15.88)	0.229	12.30 (8.38,16.82)	12.30 (7.80,15.88)	0.111
PaO_2_/FiO_2_	199.70 ± 105.10	246.20 ± 104.50	< 0.001	165.40 ± 71.61	255.20 ± 4.42	< 0.001	239.08 ± 96.05	229.76 ± 120.05	0.262
CRP	42.39 ± 5.25	41.59 ± 5.06	0.037	43.09 ± 5.30	41.58 ± 5.06	< 0.001	41.59 ± 5.06	42.39 ± 5.25	0.037
qSOFA score	1.96 ± 0.76	1.35 ± 0.62	< 0.001	1.98 ± 0.70	1.36 ± 0.64	< 0.001	1.38 ± 0.63	1.66 ± 0.77	< 0.001
SOFA score	9.58 ± 5.11	5.38 ± 4.18	< 0.001	9.21 ± 4.85	5.56 ± 4.41	< 0.001	5.66 ± 4.08	7.40 ± 5.45	< 0.001
CURB-65 score	3.16 ± 1.23	2.19 ± 1.19	< 0.001	3.29 ± 1.17	2.17 ± 1.18	< 0.001	2.22 ± 1.20	2.71 ± 1.30	< 0.001

*COPD* chronic obstructive pulmonary disease, *CVD* Cerebrovascular disease, *DM* Diabetes mellitus, *CHF* chronic heart failure, *CRD* chronic renal dysfunction, *MBP* myelin basic protein, *Lac* lactate, *PCT*: p rocalcitonin, *WBC* white blood cell, *CRP* C-reactive protein, *qSOFA* quick sequential organ failure assessment, *SOFA* sequential organ failure assessment, *CURB-65* confusion, urea, respiratory rate, blood pressure and age

In the present study, 742 CAP patients were divided into ICU (*n* = 174) and non-ICU (*n* = 568) admission groups. There were significant differences in chronic obstructive pulmonary disease (COPD) (*P* = 0.003), chronic renal dysfunction (CRD) (*P* = 0.046), MBP (*P* < 0.001), respiratory rate (*P* < 0.001), heart rate (*P* < 0.001), PaO_2_ (*P* < 0.001), Lac (*P* < 0.001), PCT (*P* < 0.001), WBC (*P* < 0.001), PaO_2_/FiO_2_ (*P* < 0.001), CRP (*P* = 0.037), qSOFA score (*P* < 0.001), SOFA score (*P* < 0.001) and CURB-65 score (*P* < 0.001) between ICU and non-ICU admission groups (Table 1).

According to CAP patients with or without ARDS, 742 cases were classified as ARDS (*n* = 164) and non-ARDS (*n* = 578) groups. Obvious differences between ARDS and non-ARDS groups were discovered in MBP (*P* < 0.001), respiratory rate (*P* = 0.002), temperature (*P* = 0.004), PaO_2_ (*P* < 0.0 01), Lac (*P* < 0.001), PCT (*P* < 0.001), PaO_2_/FiO_2_ (*P* < 0.001), CRP (*P* < 0.001), qSOFA score (*P* < 0.001), SOFA score (*P* < 0.001) and CURB-65 score (*P* < 0.001) (Table 1).

After 28 days of follow-up, we also found that the statistical differences were distinct in COPD (*P* = 0.006), DM (*P* = 0.005) tumor (*P* = 0.005), MBP (*P* < 0.001), heart rate (*P* = 0.013), Lac (*P* < 0.001), PCT (*P* < 0.001), PaO_2_/FiO_2_ (*P* < 0.001), CRP (*P* < 0.001), qSOFA score (*P* < 0.001), SOFA score (*P* < 0.001) and CURB-65 score (*P* < 0.001) between survivor (*n* = 443) and non-survivor (*n* = 299) groups (Table 1).

### Binary logistic regression analysis of the prognostic outcomes for CAP patients

As displayed in Table 2, the results showed that the differences were significant in the CRP level (*P* < 0.001, OR = 1.192, 95%CI: 1.138–1.249), Lac level (*P* < 0.001, OR = 1.227, 95%CI: 1.127–1.335), SOFA (*P* < 0.001, OR = 1.122, 95%CI: 1.063–1.184) and CURB-65 scores (*P* < 0.005, OR = 1.483, 95%CI: 1.186–1.854) on ICU-admission. It was indicated that the CRP level, Lac level, SOFA and CURB-65 scores was the risk factors of ICU-admission among CAP patients. For the occurrences of ARDS, the qSOFA (*P* = 0.034, OR = 1.605, 95%CI: 1.038–2.484), SOFA (*P* = 0.002, OR = 1.094, 95%CI: 1.034–1.159) and CURB-65 scores (*P* < 0.001, OR = 1.621, 95%CI: 1.286–2.043) were the risk factors, while the PaO_2_/FiO_2_ ratio (*P* < 0.001, OR = 0.990, 95%CI: 0.987–0.994) was as a protective factor among the patients with CAP. The Lac level (*P* < 0.001, OR = 1.138, 95%CI: 1.062–1.219) and CURB-65 scores (*P* = 0.015, OR = 1.216, 95%CI: 1.039–1.424) were the risk factors of the 28-day mortality in CAP patients.

**Table 2:** Binary Logistic regression analysis of clinical outcomes for CAP patients

	Variables	*β*	*S.E*	Wald	*P*	OR (95% CI)
ICU-admission	WBC	0.017	0.009	3.244	0.072	1.017 (0.999–1.035)
	PCT	−0.004	0.004	1.071	0.301	0.996 (0.987–1.004)
	CRP	0.176	0.024	54.278	< 0.001	1.192 (1.138–1.249)
	Lac	0.204	0.043	22.208	< 0.001	1.227 (1.127–1.335)
	PaO_2_	−0.010	0.006	3.578	0.059	0.990 (0.979–1.000)
	PaO_2_/FiO_2_	−0.001	0.002	0.476	0.490	0.999 (0.995–1.002)
	qSOFA	0.343	0.215	2.547	0.110	1.409 (0.925–2.146)
	SOFA	0.115	0.027	17.518	< 0.001	1.122 (1.063–1.184)
	CURB-65	0.394	0.114	11.956	< 0.001	1.483 (1.186–1.854)
	Constant	−10.900	1.163	87.884	< 0.001	
ARDS	WBC	−0.010	0.012	0.699	0.403	0.990 (0.967–1.014)
	PCT	0.002	0.004	0.169	0.681	1.002 (0.994–1.010)
	CRP	0.031	0.022	1.986	0.159	1.031 (0.988–1.077)
	Lac	0.029	0.041	0.511	0.475	1.030 (0.950–1.116)
	PaO_2_	−0.006	0.006	1.108	0.292	0.994 (0.983–1.005)
	PaO_2_/FiO_2_	−0.010	0.002	26.917	< 0.001	0.990 (0.987–0.994)
	qSOFA	0.473	0.223	4.519	0.034	1.605 (1.038–2.484)
	SOFA	0.090	0.029	9.661	0.002	1.094 (1.034–1.159)
	CURB-65	0.483	0.118	16.712	< 0.001	1.621 (1.286–2.043)
	Constant	−2.787	1.012	7.591	0.006	
28-day mortality	WBC	0.005	0.008	0.487	0.485	1.005 (0.990–1.020)
	PCT	0.004	0.004	1.252	0.263	1.004 (0.997–1.012)
	CRP	0.013	0.016	0.712	0.399	1.013 (0.983–1.045)
	Lac	0.129	0.035	13.619	< 0.001	1.138 (1.062–1.219)
	PaO_2_	−0.000	0.004	0.000	0.993	1.000 (0.992–1.008)
	PaO_2_/FiO_2_	0.000	0.001	0.001	0.969	1.000 (0.998–1.003)
	qSOFA	0.096	0.158	0.367	0.544	1.100 (0.808–1.499)
	SOFA	0.034	0.020	2.833	0.092	1.034 (0.994–1.075)
	CURB-65	0.196	0.081	5.901	0.015	1.216 (1.039–1.424)
	Constant	−2.251	0.706	10.171	0.001	

*WBC* white blood cell, *PCT* procalcitonin, *CR*P C-reactive protein, *Lac* lactate, *qSOFA* quick sequential organ failure assessment, *SOFA* sequential organ failure assessment, *CURB-65* confusion, urea, respiratory rate, blood pressure and age

### Prediction of the prognostic outcomes in CAP patients

**Fig. 1:**
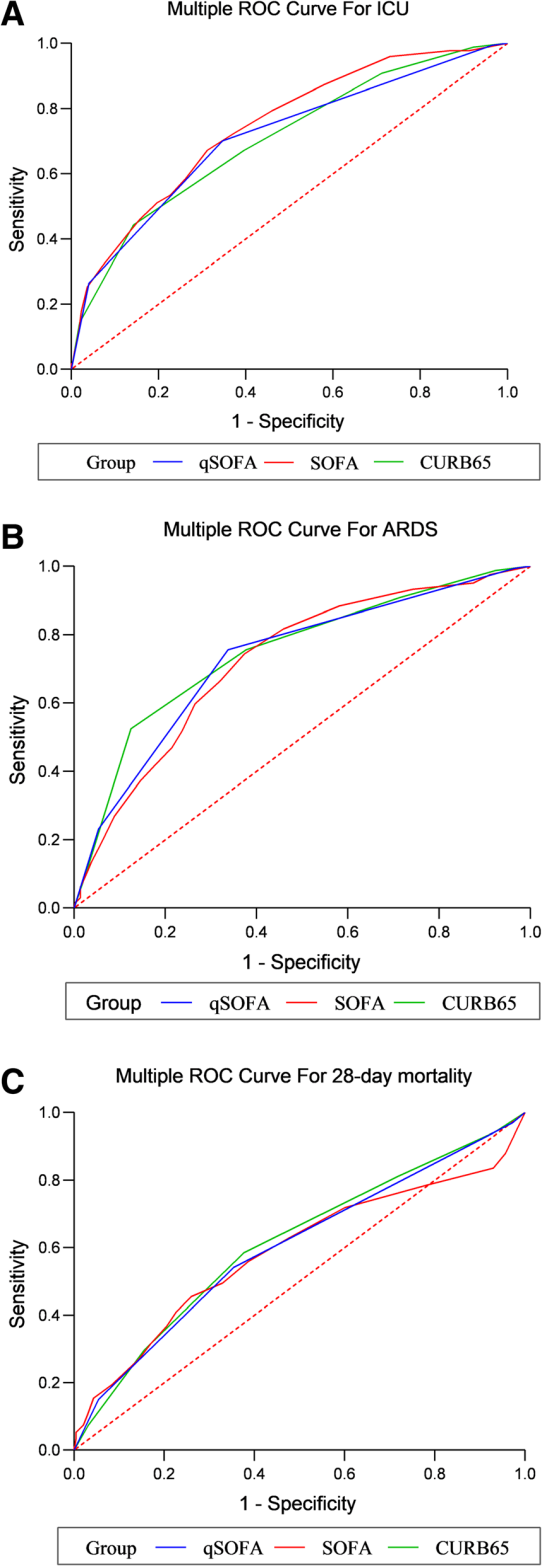
ROC curves of various predictors in prognostic outcomes in outcomes among CAP patients. **a** ICU-admission; **b** ARDS; **c** 28-day mortality

The predictive analysis of the relevant parameters for CAP patients in various outcomes were depicted in Table 3 4 and 5, and the ROC curves of predictive effects of the scoring systems for CAP patients were shown in Fig. 1. The AUC values of the qSOFA, SOFA and CURB-65 scores for ICU-admission among CAP patients were 0.712 (95%CI: 0.678–0.745, *P* < 0.001), 0.744 (95%CI: 0.711–0.775, *P* < 0.001) and 0.705 (95%CI: 0.671–0.738, *P* < 0.001), respectively. The cut-off values for the qSOFA, SOFA and CURB-65 scores maximizing the composite of specificity and sensitivity in the prediction of CAP patients in ICU-admission were 1.0, 6.0 and 3.0 (Table 3 and Fig. 1a). For ARDS, the AUC values of the qSOFA, SOFA and CURB-65 scores were 0.730 (95%CI: 0.697–0.762, *P* < 0.001), 0.724 (95%CI: 0.690–0.756, *P* < 0.001) and 0.749 (95%CI: 0.716–0.780, *P* < 0.001), respectively (Table 4 and Fig. 1b). After 28 days of follow-up, the AUC values of the qSOFA, SOFA and CURB-65 scores for 28-day mortality were 0.602 (95%CI: 0.566–0.638, *P* < 0.001), 0.587 (95%CI: 0.551–0.623, *P* < 0.001) and 0.614 (95%CI: 0.577–0.649, *P* < 0.001) in turn (Table 5 and Fig. 1c).

**Table 3:** The characteristics for various predictors of ICU-admission in CAP patients

Variables	AUC (95% CI)	*S.E*	*P*	Cut off	Sensitivity	Specificity	PPV	NPV	LR+	LR-
Lac	0.745 (0.712–0.776)	0.022	< 0.001	2.3	64.37	75.18	43.3	87.3	2.59	0.47
PCT	0.641 (0.605–0.676)	0.023	< 0.001	1.0	50.00	73.98	37.2	82.8	1.92	0.68
WBC	0.563 (0.527–0.599)	0.027	0.019	18.48	28.16	85.21	36.8	79.5	1.90	0.84
CRP	0.721 (0.687–0.753)	0.023	< 0.001	44.01	59.77	75.53	42.8	86.0	2.44	0.53
qSOFA	0.712 (0.678–0.745)	0.021	< 0.001	1.0	70.11	65.32	38.2	87.7	2.02	0.46
SOFA	0.744 (0.711–0.775)	0.021	< 0.001	6.0	67.24	68.84	39.8	87.3	2.16	0.48
CURB-65	0.705 (0.671–0.738)	0.022	< 0.001	3.0	44.25	85.74	48.7	83.4	3.10	0.65

*Lac* lactate, *PCT* procalcitonin, *WBC* white blood cell, *CRP* C-reactive protein, *qSOFA* quick sequential organ failure assessment, *SOFA* sequential organ failure assessment, *CURB-65* confusion, urea, respiratory rate, blood pressure and age

**Table 4:** The characteristics for various predictors of ARDS in CAP patients

Variables	AUC (95% CI)	*S.E*	*P*	Cut off	Sensitivity	Specificity	PPV	NPV	LR+	LR-
Lac	0.690 (0.655–0.723)	0.023	< 0.001	2.1	61.59	69.20	36.2	86.4	2.00	0.56
PCT	0.662 (0.627–0.696)	0.023	< 0.001	0	68.29	59.48	32.5	86.8	1.69	0.53
WBC	0.531 (0.494–0.567)	0.027	0.260	12.6	51.83	58.48	26.2	81.1	1.25	0.82
CRP	0.587 (0.550–0.622)	0.025	0.001	40.8	71.34	42.91	26.2	84.1	1.25	0.67
qSOFA	0.730 (0.697–0.762)	0.021	< 0.001	1.0	75.61	66.26	38.9	90.5	2.24	0.37
SOFA	0.724 (0.690–0.756)	0.022	< 0.001	5.0	74.39	62.63	36.1	89.6	1.99	0.41
CURB-65	0.749 (0.716–0.780)	0.022	< 0.001	3.0	52.44	87.54	54.4	86.6	4.21	0.54

*Lac* lactate, *PCT* procalcitonin, *WBC* white blood cell, *CRP* C-reactive protein, *qSOFA* quick sequential organ failure assessment, *SOFA* sequential organ failure assessment, *CURB-65* confusion, urea, respiratory rate, blood pressure and age

**Table 5:** The characteristics for various predictors of 28-day mortality in CAP patients

Variables	AUC (95% CI)	*S.E*	*P*	Cut off	Sensitivity	Specificity	PPV	NPV	LR+	LR-
Lac	0.611 (0.575–0.646)	0.022	< 0.001	2.3	45.48	73.59	53.8	66.7	1.72	0.74
PCT	0.624 (0.588–0.659)	0.020	< 0.001	0	59.80	62.08	51.3	69.8	1.58	0.65
WBC	0.534 (0.498–0.571)	0.022	0.115	11.68	54.85	54.63	44.9	64.2	1.21	0.83
CRP	0.537 (0.501–0.574)	0.022	0.083	45.3	28.09	78.56	46.9	61.8	1.31	0.92
qSOFA	0.602 (0.566–0.638)	0.019	< 0.001	1.0	54.18	64.56	50.8	67.6	1.53	0.71
SOFA	0.587 (0.551–0.623)	0.022	< 0.001	7.0	45.48	74.04	54.2	66.8	1.75	0.74
CURB-65	0.614 (0.577–0.649)	0.021	< 0.001	2.0	58.53	62.30	51.2	69.0	1.55	0.67

*Lac* lactate, *PCT* procalcitonin, *WBC* white blood cell, *CR*P C-reactive protein, *qSOFA* quick sequential organ failure assessment, *SOFA* sequential organ failure assessment, *CURB-65* confusion, urea, respiratory rate, blood pressure and age

Comparison of AUCs for predicting the prognostic outcomes among CAP patients were analyzed in the study. The results revealed that there were no statistical differences between qSOFA and SOFA scores for predicting ICU-admission (Z = 1.482, *P* = 0.138), ARDS (Z = 0.321, *P* = 0.748) and 28-day mortality (Z = 0.573, *P* = 0.567). Moreover, we found no differences to predict the ICU-admission (Z = 0.370, *P* = 0.712), ARDS (Z = 0.900, *P* = 0.368) and 28-day mortality (Z = 0.768, *P* = 0.442) using qSOFA or CURB-65 scores.

## Discussion

CAP is one of serious diseases that causes death and threatens human life. Establishment of safe and effective prognostic assessment systems of CAP is important for clinicians. In the present study, we investigated the predictive value of qSOFA for the clinical outcomes including ICU-admission, ARDS and 28-day mortality in emergency patients with CAP, and the efficacy of the qSOFA score was assessed in comparison with CURB-65 and SOFA scores. Our results revealed that qSOFA was not inferior to SOFA or CURB-65 scores in predicting the ICU-admission, ARDS and 28-day mortality of patients presenting in the ED with CAP.

Pneumonia is a common reason for hospitalization [18], and CAP is associated with the high risk of respiratory failure or septic organ dysfunction. The early managements of CAP are also based on severity assessment tools since 2000, including CURB-65 [19–21], pneumonia severity index (PSI) [21–23] and so on. In 2007, the Infectious Diseases Society of America/American Thoracic Society consensus guidelines suggested PSI and CURB-65 scoring systems be used together [24]. Subsequently, newly published guidelines suggested that the pneumonia severity can be assessed utilizing the SOFA and qSOFA scores. Nevertheless, the efficacies of these scoring systems for predicting the prognostic outcomes are still matters of controversy in emergency patients with CAP. As we know, the calculation of PSI is really complex that involving 20 parameters [22], which is not suitable for newly admitted patients in ED. The CURB-65 score consists of confusion, urea, respiratory rate, blood pressure and age that is similar with the qSOFA score in components. The SOFA score was proposed by European Society of Intensive Care Medicine (ESICM) in 1994 [25], and was applied for prognostic assessments in sepsis and multiple organ dysfunction, which was associated with the mortality. The qSOFA, incorporating hypotension, altered mental status and tachypnea, was high prediction of mortality in non-ICU settings [26]. In our study, the qSOFA, SOFA and CURB-65 scoring systems were applied for mortality prediction in patients with CAP.

Our findings found that the predictive performances of the qSOFA and CURB-65 scores in ICU-admission, ARDS and 28-day mortality were similar (*P* > 0.05). The qSOFA score is a quick and simple scoring system which is to identify the elevated risks in clinical deterioration, which was associated with ICU-admission in adult ED patients [27]. Goulden et al [28] reported that the efficacy of the qSOFA was as similar as Systemic Inflammatory Response Syndrome (SIRS) and National Early Warning Score (NEWS) for predicting ICU-admission. Early researches mentioned that a positive qSOFA score had high specificity outside the ICU in early detection of ICU admission [14]. Previous studies reported that the CURB-65 score was related to the risk of ARDS in CAP patients, and the development of ARDS risk was prominent when the CURB-65 score was ≥2 [29]. The CURB-65 score contains respiratory rate ≥ 30 beats/min and systolic blood pressure < 90 mmHg or diastolic blood pressure ≤ 60 mmHg, which the thresholds of these indicators were higher than that in qSOFA (respiratory rate ≥ 22 beats/min and systolic blood pressure ≤ 100 mmHg [13]). Compared with the qSOFA score, CURB-65 may miss some potentially dangerous patients in the risk prediction. A recent systematic review suggested that the positive qSOFA score had high specificity in early detection of in-hospital mortality [14]. A multiple-center research discovered that the CURB-65 score had a predictive value of mortality in CAP patients [30]. In the presented study, the predictive values of the qSOFA and CURB-65 scores were similar regarding to the ICU-admission, ARDS and 28-day mortality. The discrepancy may be due to differences in the variables assessed, the patient group and the study design. In consideration of needing quick and effective diagnose in the ED, therefore the qSOFA score may be a useful and practical tool for the early prediction of ARDS and 28-day morality among patients with CAP, and may serve as an early warning signal that CAP is about to worsen in emergency patients.

The superiority of this study was that few previous researches had investigated the predictive value of qSOFA score in comparison with other pneumonia severity scoring systems (SOFA and CURB-65 scores) in the prognostic outcomes containing ICU-admission, ARDS and 28-day mortality for CAP patients, especially in Chinese population. However, there were some limitations that should be warranted caution for interpreting the data. Our investigation was a retrospective study based on a single center. Microbiology, time to initiation of antibiotics and antibiotic choice may be potential confounding variables. The 28-day mortality as a prognostic outcome was assessed, but long-term outcomes were not identified. Thus, prospective multicenter studies with larger samples should be needed for further verification in clinic.

## Conclusion

In this study, we found that the predictive effects of qSOFA score were similar with CURB-65 and SOFA scores in ICU-admission, ARDS and 28-day mortality. According to the actual situation of emergency patients, qSOFA score may be an effective and practical tool for the early prediction of ICU-admission, ARDS and 28-day morality among CAP patients in the ED.

## Data Availability

The data used and/or analyzed during the current study are available from the corresponding author on reasonable request.

## Abbreviations

qSOFA: Quick sequential organ failure assessment
CAP: Community-acquired pneumonia
ED: Emergency department
ARDS: Acute respiratory distress syndrome
AUC: Area under the curve
ICU: Intensive care unit
IMV: Invasive mechanical ventilation
EDTA: Ethylenediaminetetraacetate
WBC: White blood cell
PCT: Procalcitonin; LOD limit of detection
CRP: C-reactive protein
SD: Standard deviation
AUC: Area under curves
PPV: Positive predictive value
NPV: Negative predictive value
COPD: Chronic obstructive pulmonary disease
CFD: Chronic renal dysfunction
PSI: Pneumonia severity index
ESICM: European Society of Intensive Care Medicine
SIRS: Systemic Inflammatory Response Syndrome
NEWS: National Early Warning Score

## References

[1] Musher DM, Thorner AR (2014). Community-acquired pneumonia. N Engl J Med.

[2] Welte T, Torres A, Nathwani D (2012). Clinical and economic burden of community-acquired pneumonia among adults in Europe. Thorax..

[3] Leoni D, Rello J (2017). Severe community-acquired pneumonia: optimal management. Curr Opin Infect Dis.

[4] Fan E, Brodie D, Slutsky AS (2018). Acute respiratory distress syndrome advances in diagnosis and treatment. JAMA.

[5] Song H, Moon HG, Kim SH (2019). Efficacy of quick sequential organ failure assessment with lactate concentration for predicting mortality in patients with community-acquired pneumonia in the emergency department. Clin Exp Emerg Med.

[6] Ranzani OT, Prina E, Menendez R, Ceccato A, Cilloniz C, Mendez R (2017). New sepsis definition (Sepsis-3) and community-acquired pneumonia mortality a validation and clinical decision-making study. Am J Respir Crit Care Med.

[7] Kim MW, Lim JY, Oh SH (2017). Mortality prediction using serum biomarkers and various clinical risk scales in community-acquired pneumonia. Scand J Clin Lab Investig.

[8] Kofoed K, Eugen-Olsen J, Petersen J, Larsen K, Andersen O (2008). Predicting mortality in patients with systemic inflammatory response syndrome: an evaluation of two prognostic models, two soluble receptors, and a macrophage migration inhibitory factor. Eur J Clin Microbiol Infect Dis.

[9] Ilg A, Moskowitz A, Konanki V, Patel PV, Chase M, Grossestreuer AV (2019). Performance of the CURB-65 score in predicting critical care interventions in patients admitted with community-acquired pneumonia. Ann Emerg Med.

[10] Espana PP, Capelastegui A, Mar C, Bilbao A, Quintana JM, Diez R (2015). Performance of pro-adrenomedullin for identifying adverse outcomes in community-acquired pneumonia. J Infect.

[11] Lim WS, van der Eerden MM, Laing R, Boersma WG, Karalus N, Town GI (2003). Defining community acquired pneumonia severity on presentation to hospital: an international derivation and validation study. Thorax..

[12] Espana PP, Capelastegui A, Gorordo I, Esteban C, Oribe M, Ortega M (2006). Development and validation of a clinical prediction rule for severe community-acquired pneumonia. Am J Respir Crit Care Med.

[13] Singer M, Deutschman CS, Seymour CW, Shankar-Hari M, Annane D, Bauer M (2016). The third international consensus definitions for sepsis and septic shock (Sepsis-3). JAMA.

[14] Song JU, Sin CK, Park HK, Shim SR, Lee J. Performance of the quick sequential (sepsis-related) organ failure assessment score as a prognostic tool in infected patients outside the intensive care unit: a systematic review and meta-analysis. Crit Care. 2018;22:28–40. 10.1186/s13054-018-1952-x PMC5802050 29409518

[15] Asai N, Watanabe H, Shiota A, Kato H, Sakanashi D, Hagihara M (2019). Efficacy and accuracy of qSOFA and SOFA scores as prognostic tools for community-acquired and healthcare-associated pneumonia. Int J Infect Dis.

[16] Niederman MS, Mandell LA, Anzueto A, Bass JB, Broughton WA, Campbell GD (2001). Guidelines for the management of adults with community-acquired pneumonia - diagnosis, assessment of severity, antimicrobial therapy, and prevention. Am J Respir Crit Care Med.

[17] Ranieri VM, Rubenfeld GD, Thompson BT, Ferguson ND, Caldwell E, Fan E (2012). Acute respiratory distress syndrome the Berlin definition. JAMA.

[18] WHO Global Health Observatory (GHO). http://www.who.int/gho/mortality_ burden_disease/causes_death_2008/en/index.html; Accessed 2018.

[19] British Thoracic Society Standards of Care Committee (2001). BTS guidelines for the management of community acquired pneumonia in adults-synopsis. Thorax..

[20] Capelastegul A, Espana PP, Quintana JM, Areltio I, Gorordo I, Egurrola M (2006). Validation of a predictive rule for the management of community-acquired pneumonia. Eur Respir J.

[21] Chen JH, Chang SS, Liu JJ, Chan RC, Wu JY, Wang WC (2010). Comparison of clinical characteristics and performance of pneumonia severity score and CURB-65 among younger adults, elderly and very old subjects. Thorax..

[22] Fine MJ, Auble TE, Yealy DM, Hanusa BH, Weissfeld LA, Singer DE (1997). A prediction rule to identify low-risk patients with community-acquired pneumonia. N Engl J Med.

[23] Buising KL, Thursky KA, Black JF, MacGregor L, Street AC, Kennedy MP (2006). A prospective comparison of severity scores for identifying patients with severe community acquired pneumonia: reconsidering what is meant by severe pneumonia. Thorax..

[24] Mandell LA, Wunderink RG, Anzueto A, Bartlett JG, Campbell GD, Dean NC (2007). Infectious Diseases Society of America/American Thoracic Society consensus guidelines on the management of community-acquired pneumonia in adults. Clin Infect Dis.

[25] Vincent JL, Moreno R, Takala J, Willatts S, DeMendonca A, Bruining H (1996). The SOFA (sepsis-related organ failure assessment) score to describe organ dysfunction/failure. Intensive Care Med.

[26] Seymour CW, Liu VX, Iwashyna TJ, Brunkhorst FM, Rea TD, Scherag A (2016). Assessment of clinical criteria for sepsis for the third international consensus definitions for sepsis and septic shock (Sepsis-3). JAMA.

[27] Singer AJ, Ng J, Thode HC, Spiegel R, Weingart S (2017). Quick SOFA scores predict mortality in adult emergency department patients with and without suspected infection. Ann Emerg Med.

[28] Goulden R, Hoyle MC, Monis J, Railton D, Riley V, Martin P (2018). qSOFA, SIRS and NEWS for predicting inhospital mortality and ICU admission in emergency admissions treated as sepsis. Emerg Med J.

[29] Liu KT, Yang KY, Lee YC, Perng RP (2007). Risk factor analysis of acute respiratory distress syndrome among hospitalized patients with chlamydophila pneumoniae pneumonia. J Chin Med Assoc.

[30] Ewig S, Birkner N, Strauss R, Schaefer E, Pauletzki J, Bischoff H (2009). New perspectives on community-acquired pneumonia in 388406 patients. Results from a nationwide mandatory performance measurement programme in healthcare quality. Thorax..

